# Inhalation of Nebulized Fluids

**Published:** 1866-07

**Authors:** 


INHALATION OF NEBULZIED FLUIDS.

Dr. J. Solis Cohen, of Philadelphia, read a paper entitled "Remarks on the Inhalation of Nebulized Liquids, and their Topical Application to Inflamed and Ulcerated Surfaces."

The reader stated that his design was not so much to present a review of the knowledge of the profession on these topics, or to bring forward any of his peculiar views, as much as to give a resumé of the results thus far obtained from recent experience, with a view of eliciting upon a subsequent occasion a valuable paper on the whole subject of the Therapeutics of Inhalation, which should embody the experience of a number of observers, and furnish the profession with authoritative data for their general investigation, inasmuch as the literature on this subject, especially in this country, was very meagre and unsatisfactory.

The paper stated that the idea of treating diseases of the respiratory organs by inhalation, which should bring remedies directly in contact with diseased surfaces, was as old as the healing art itself; for what was more natural than to imitate the entrance of the vivifying breath of life in its access to the economy? The records of classic Greece and Rome show that this principle was taken advantage of in the inhalation of volatile substances, and in the residence of patients upon the seashore, and in the neighborhood of volcanoes. This method of treatment fell into disuse, and was again resorted to many times, and had fallen into permanent neglect for a long period, until the discoveries of oxygen leading to further chemical research, and the promulgation of the exhilarating effects following inhalation of the nitrous oxide gas, again directed attention to the subject of inhalation as a remedial agent. Then the ammoniacal exhalations from cattle-yards, stables, etc., became largely employed in many affections, until too extended empirical application to all the ills of life meeting with failure, led the whole system into disuse, even for these affections for which it was originally intended. The discoveries of the properties chlorine, iodine, etc., of the effects of the smoking of opium among the Chinese, led to the employment of similar substances; and the whole series of narcotics, resins, and all substances producing vapor became employed in this manner.

Observations of the diseases affecting workers in minerals, metals, etc., and the *post mortem* revelations of the scalpel, proving the entrance of particles of dust into the lungs, led to the therapeutic employment of powders by inhalation.

After some other general remarks of a similar nature, the paper went on to relate that within a few years a new method of presenting *liquid substances* for inhalation *as such*, had become much employed in Europe, and seemed to promise an extensive field of success in the treatment of the whole class of pectoral troubles.

Every one is familiar with the effect of a stream of water directed upon the side of a house, how a large portion of the volume of water is directed back in a shower of small streams, or coarse spray. In certain bathing establishments in Germany, many liquid applications are made on this principle to the surface of the body in the treatment of affections of special organs, and of the general system likewise. Streams of the medicated liquid are forced against metallic plates on the walls of the bathing apartments, and fall over the desired surfaces in this shower of coarse spray.

It having been noticed that amelioration of concomitant chest affections seemed to follow this exposure for other purposes, patients were sent to these establishments to respire the air thus impregnated. The beneficial results obtained at the Inhalatorium of Dr. Auphan, of Eaux-les-Bains, established in

1849, led to similar establishments elsewhere, until finally, Sales-Girons, who in connexion with Flubé had in 1856 erected an inhalatorium at Pierre-fonds, conceived the idea of constructing on this principle a portable apparatus which could be employed by patients at their residences, and thus admit of much wider application. Sales-Girons exhibited an instrument of this kind to the Academy in 1858. His apparatus consisted of a condensing syringe, which forced a very fine stream of liquid against a convex metallic button, converting it in deflection into a very finely divided spray, having much the appearance of a dense fog or mist, the separate particles of which, suspended as they are for a time in the atmosphere playing around much like the dust observed in a sumbeam, can be drawn into the lungs by inspiration.

The paper referred to several of these apparatus, especially the modification of the tubes of Bergson, and the employment of steam by Siegle, in lieu of the compressed air, and stated that experiments upon living animals proved the entrance of these particles into the air-cells; though for various reasons, the spray striking against many surfaces during its ingress, its dissipation into a much larger volume than could be received into the mouth, etc., but a small quantity of the liquid thus nebulized (the writer thought not more than twelve per cent), could reach the smaller bronchial ramifications.

The writer thought that the application in this manner of liquid substances of known strength directly to the diseased surfaces would prove more prompt in effect, whether for good or ill, than when the entire current of the circulation became the vehicle of communication, perhaps affecting the general system when not desirable. The mucous membrane of the lungs is quick to absorb, secretes no chemical product to modify chemical medicines, and has no temporary ingesta to interfere with the direct action of remedial agents applied to it.

Although the results thus far obtained are far from being positive, and many of them unsatisfactory and negative, still they are such as to justify careful research into this new method of medication.

Some conclusions were then given as the results of recorded experience; the results of the writer's personal experience and observations, and the results of extensive experiments, carefully conducted by Dr. J. M. Da Costa, at the Pennsylvania Hospital, as well as those obtained by the latter named gentleman in his private practice.

A few remarks followed as to the manner of preparing medicines for employment in this way, and their application when different parts of the air-passages were to be medicated.

Allusion was made to the employment of this system of nebulization for local applications to inflamed and ulcerated surfaces on the exterior of the body, and a certain distance within the various cavities, and an instrument devised for this purpose was exhibited, to demonstrate that in a second or two a layer of a caustic or other solution, many times more delicate than any film which could be deposited by the slightest touch of a hair pencil, could be directed upon an ulcerated or inflamed surface, whether of large or small surface. A continuous application of course can be kept up when desired, and in this connexion the method was recommended for the production of local anæsthesia for minor operations in surgery—a subject at present exciting a good deal of attention, and brought to the notice of the profession by Dr. B. W. Richardson, of London.

On motion, the paper was referred to the Committee on Publication, for publication in the Transactions of the year; but at the request of the writer the motion was modified to call for the appointment of a special committee to report on the "Therapeutics on Inhalation," at the next annual meeting of the Association.

On motion, the paper was received.

Dr. J. E. Clawson, of Delaware, moved that the Section appoint a Special Committee on the Therapeutics of Inhalation, to consist of three members, and that the paper of Dr. J. Solis Cohen, just read, be referred to that Committee. Carried.

The Chairman appointed as such Committee:—

Drs. J. Solis Cohen, Philadelphia; J. M. DaCosta, Philadelphia; L. Elsberg, New York.

Adjourned.

THIRD SESSION.

The Section met at Concordia Hall, on Thursday, May 3, 1866, at 1.45 P.M.

In the absence of the chairman, Dr. Ellsworth Eliot, of New York, was elected Chairman *pro tem.*

The minutes of the last meeting having been read and approved, the Section adjourned *sine die.*

